# Features of animal babbling in the vocal ontogeny of the gray mouse lemur (*Microcebus murinus)*

**DOI:** 10.1038/s41598-023-47919-7

**Published:** 2023-12-04

**Authors:** Alexandra Langehennig-Peristenidou, Daniel Romero-Mujalli, Tjard Bergmann, Marina Scheumann

**Affiliations:** 1 Institute of Zoology, University of Veterinary Medicine Hannover, Bünteweg 17, 30559 Hannover, Germany; 2 Department for Environment Constructions and Design, Institute of Microbiology (IM), University of Applied Sciences and Arts of Southern Switzerland (SUPSI), 6850 Mendrisio, Switzerland

**Keywords:** Zoology, Animal behaviour

## Abstract

In human infants babbling is an important developmental stage of vocal plasticity to acquire maternal language. To investigate parallels in the vocal development of human infants and non-human mammals, seven key features of human babbling were defined, which are up to date only shown in bats and marmosets. This study will explore whether these features can also be found in gray mouse lemurs by investigating how infant vocal streams gradually resemble the structure of the adult trill call, which is not present at birth. Using unsupervised clustering, we distinguished six syllable types, whose sequential order gradually reflected the adult trill. A subset of adult syllable types was produced by several infants, with the syllable production being rhythmic, repetitive, and independent of the social context. The temporal structure of the calling bouts and the tempo-spectral features of syllable types became adult-like at the age of weaning. The age-dependent changes in the acoustic parameters differed between syllable types, suggesting that they cannot solely be explained by physical maturation of the vocal apparatus. Since gray mouse lemurs exhibit five features of animal babbling, they show parallels to the vocal development of human infants, bats, and marmosets.

## Introduction

To acquire their maternal language, human infants undergo four stages of language development in their first year of life^1–3^. Starting with the phonation stage (from birth until the second month of life), human infants emit quasi-vowels, which are produced without the typical position of the tongue, jaw, and lips for the respective vowel (e.g.,^2,4^). Subsequently, the primitive articulation phase follows (two to four months of life), where infants start emitting their first articulated sounds termed gooing. In the expansion stage (three to eight months), infants exhibit marginal babbling. Thereby, a consonant-like sound (e.g., m, d) is combined with a full vowel-like sound (e.g., a) to a first protophone syllable (e.g., ma, da). Concurrently, infants start to intensively play with their voices, showing a high rate of protophones even when they are alone^1,3^. In the canonical babbling stage repetitions of these protophone syllables are observed, resulting in a rapid transition between consonant and vowel-like sounds similar to human speech (e.g., mama, dada). While deaf-human infants do not overcome the pre-canonical stage^5–7^, hearing infants learn the association between the protophone syllables and the meaning by the auditory feedback of the caregivers^2,8,9^, transferring them to words (e.g., baba dada daddy)^2^. Thus, the rapid increase and combination of protophone syllables builds the basis for the auditory-feedback dependent acquisition of maternal language.

Investigating parallels between human babbling and the vocal development in non-human mammals, Elowson et al.^10^ and Fernandez et al.^11^ defined seven key features of human babbling: (1) *Universality* (i.e., protophones produced by infants irrespective of their cultural background), (2) *Syllable subset acquisition* (i.e., includes phonetic units found in adult speech), (3) *Independence of social context* (i.e., occurs also in the absence of a vocal referent and lacks apparent meaning), (4) *Rhythmic and repetitive* (i.e., rhythmic phonation and repetition of canonical syllables), (5) *Babbling bout composition* (i.e., combination of consonant and vowel units into sequences), (6) *Peak of high vocal plasticity* (i.e., early onset of babbling with a later peak of emergence of syllable types), (7) *Facilitating caregiver interactions* (i.e., used during oscillating parent-infant interactions). Using these features, similarities between human babbling and the vocal ontogeny of marmosets (e.g.,^10,12–16^) and bats (e.g.,^3,11,17^) have been shown, which we refer here as animal babbling. Thereby, in marmosets and bats, infants from different populations produce similar acoustic features included in the adult vocal behavior^10,11^. These infant vocalizations do not serve a communicative function, with them also being produced in the absence of a vocal referent, indicating vocal play^10,11^. In addition, the vocal streams are rhythmical and repetitive, gradually resembling adult well-formed vocal units (“canonical syllables”)^10,11^. Thereby, they facilitate caregiver interactions^8,10,13,18–20^. Furthermore, a peak of high vocal plasticity is documented in bats^11^ but controversial results were found in marmosets: whereas Takahashi et al.^16^ report that immature phee calls disappear in adulthood, Gultekin et al.^21^ documented a continued high vocal plasticity even in adult stages. Even though vocal plasticity during infancy is reported for several non-human mammals (e.g.,^22–27^), evidence for animal babbling is rare (see for review^3^).

While auditory-feedback is necessary for human infants to overcome the pre-canonical stage^8,9^, for animal babbling it is critically discussed whether it is a result of auditory-feedback or physical maturation^28^. To date, auditory-feedback dependent gradual acquisition of adult vocalizations has been described for some bat species (e.g.,^22–24^), while parental vocal feedback accelerates the acquisition of adult vocalizations in common marmosets (e.g.,^8,13,18–20,29^). Nevertheless, age-dependent changes in the tempo-spectral parameters can be a result of physical maturation. For example, a growing lung capacity can explain an increase in the duration of single syllables or even whole calls, while the increase in body size can result in a lower fundamental frequency^21^. However, Gultekin et al.^21^ argued that even though age-dependent changes in the tempo-spectral acoustic parameters of the syllables may be a result of physical maturation, the variable sequences and transitions of syllable types within a vocal stream might represent a vocal plasticity comparable to human infant vocal behavior. Recently, variable vocal streams during infancy were also reported for an additional primate species, the gray mouse lemur (*Microcebus murinus*)^30^. Adult gray mouse lemurs produce a complex advertisement call, which is not present at birth, and show the ability for vocal convergence^31^. Thus, they constitute a new promising animal model investigating animal babbling.

Gray mouse lemurs (*Microcebus murinus*) are small, nocturnal, wet-nosed primates endemic to Madagascar^32^. Females give birth to one to four infants, which are blind until the 6^th^ day of life but are born with an open ear canal^33–35^. In contrast to other primates, gray mouse lemurs evolved an infant-parking system with the mother leaving the infants on their own during foraging^36^. At around three weeks, gray mouse lemur infants start leaving the nest box on their own, while they are weaned between 25 and 35 days^33^. Gray mouse lemurs possess a broad vocal repertoire. The adults emit at least ten different call types, with the majority of the vocalizations being ultrasonic (e.g.,^37,38^). The most complex call type is the trill call uttered during mating, forming kin-related sleeping groups or by the mother during mother-infant reunions (e.g.,^30,39,40^). A trill consists of up to 30 syllables, which are distinguished into three structural parts: (1) the first part, a variable initial modulation, (2) the middle part comprising of down and/or up modulated syllables and (3) the end part consisting of syllables of almost constant frequency^31,33,41^. The trill call is not emitted by the infants directly after birth, while precursors of the trill termed prototrills have been observed, suggesting that the trill call develops during infancy^30,33,42^.

Aim of this study is to investigate to which extent gray mouse lemur infants demonstrate features of animal babbling. Therefore, (1) we investigate the ontogenetic development of infant syllables, (2) we test whether syllable types are produced in isolation contexts or require an external referent, (3) we investigate how the sequential order of the syllables in a calling bout changes throughout the age classes and (4) how the temporal structure of the calling bouts and the spectro-temporal structure of syllable types changes with age. Thereby, a comparison with the adult trill calls is to be carried out, to test whether the calling bouts gradually resemble the adult trill calls in the aforementioned aspects.

## Results

### Definition of syllable types using unsupervised cluster analysis

**Figure 1:**
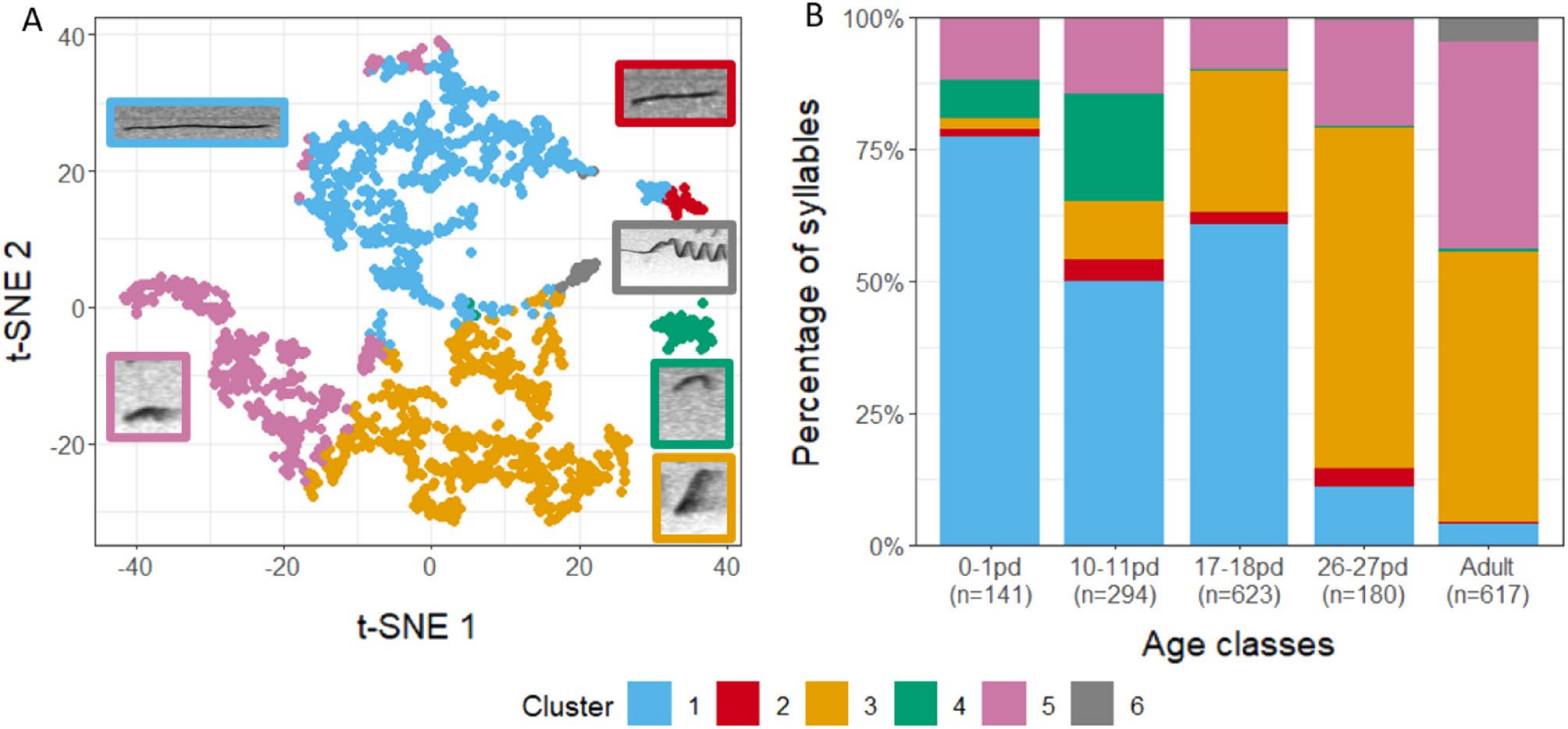
Syllable types obtained by the unsupervised cluster analysis. (**A**) Visualization of the clusters acquired by the K-means cluster analysis by plotting function 1 and 2 of a t-SNE analysis. Each color corresponds to a different cluster, with an example of the contour of the respective syllable type annotated in the same color. The examples of the syllable contours are illustrated matching to the length of the respective syllable, thus, durations are not proportional in sizes. (**B**) Composition of clusters for each age class. pd = postnatal day; n = number of syllables.

**Figure 2:**
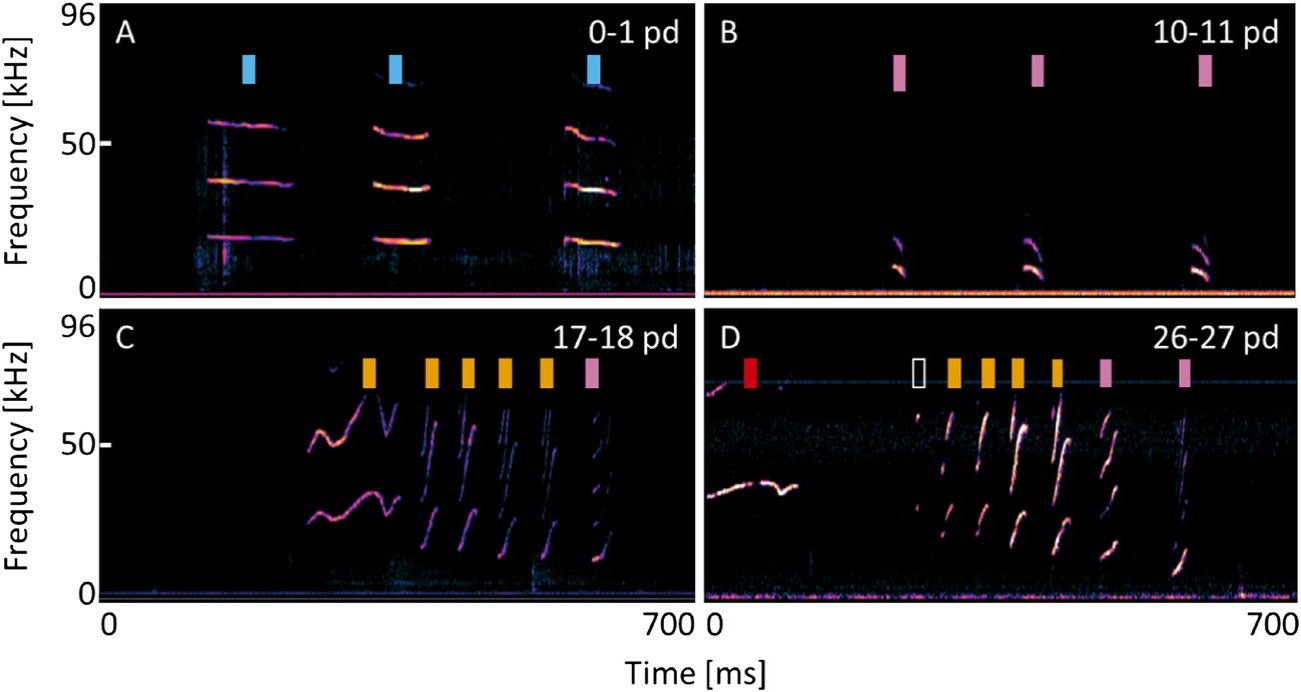
Spectrograms of calling bouts produced by infants in all four infant age classes. (**A**) 0–1 postnatal days (pd), (**B**) 10–11 pd, (**C**) 17–18 pd, (**D**) 26–27 pd; Colored rectangles represent assigned clusters: blue = Cluster 1; pink = Cluster 5; orange = Cluster 3; red = Cluster 2; empty rectangle = syllable was not included in the analysis due to low quality; pd = postnatal day.

**Figure 3:**
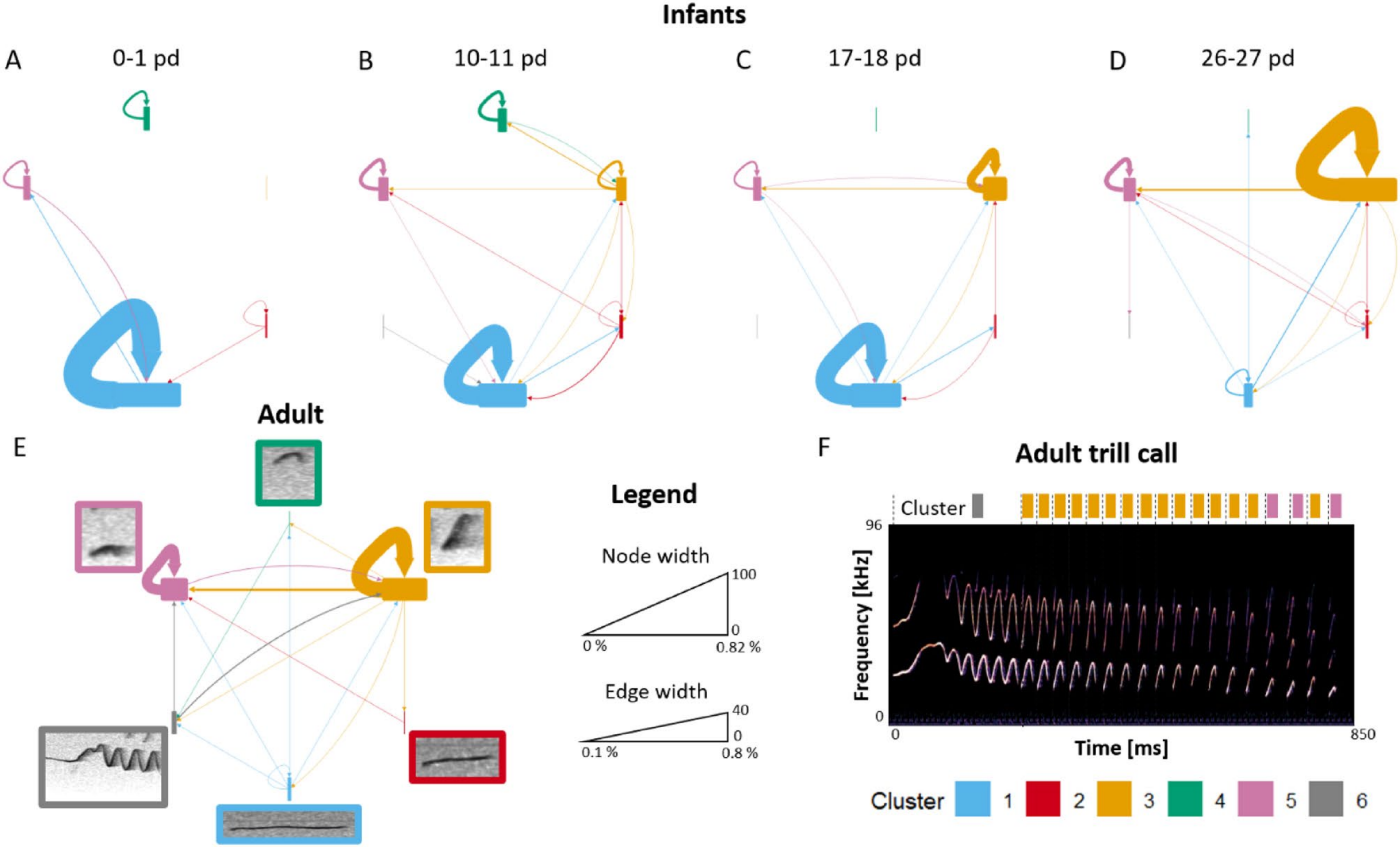
Syntactic structure per age class (**A**) 0–1 postnatal days (pd), (**B**) 10–11 pd, (**C**) 17–18 pd, (**D**) 26–27 pd, (**E**) Adults with (**F**) a spectrogram of an adult trill call. Networks: The edges are directional, being colored in the same color as the previous syllable of a transition. Repetitions of the same syllable type are indicated by self-loops. The width of the nodes and the edges are proportional to the percentage of the transition in the respective age class. Spectrogram: Dash line marks the start of the syllable. Colored rectangle on the top marks the assigned cluster. pd = postnatal day.

We measured 10 acoustic parameters to describe the tempo-spectral structure of 1238 syllables of 266 infant calling bouts recorded during four developmental stages (0–1 postnatal days (pd)—birth; 10–11 pd—nursing period; 17–18 pd—infants start leaving the nests on their own; 26–27 pd—weaning^33^) and additional 617 syllables of 50 adult trills as reference calls. To avoid an observational bias in syllable classification, we performed an unsupervised cluster analysis using the following four non-correlating acoustic parameters: syllable duration, percentage of voiced frames, mean fundamental frequency and standard deviation of fundamental frequency (Fig. S1). Applying the K-means clustering algorithm with the knee method to identify the ideal number of clusters resulted to the syllables being separated into six clusters representing six different syllable types (Fig. 1, Fig. S2; Acoustic description Tab. S1). Visualizing the clusters using the t-SNE method supported the separation of the clusters (Fig. 1A). Cluster 1 (n = 679, N_infants/families_ = 21, N_adults_ = 14, Figs. 1A, 2A) contained flat syllables (bandwidth: 2.3 ± 1.5 kHz) with an average length of 56 ± 28 ms. Cluster 2 (n = 36, N_infants/families_ = 13, N_adults_ = 2, Figs. 1A, 2D) contained syllables with an average length of 49 ± 49 ms, a mean frequency of around 20 kHz (22.1 ± 4.3 kHz) and a bandwidth of 5.1 ± 4.4 kHz. Cluster 3 (n = 634, N_infants/families_ = 8, N_adults_ = 31) comprised of syllables with an average length of 24 ± 19 ms, high bandwidth and variable modulation (bandwidth: 10.0 ± 2.8 kHz). Thereby, syllables could show single or multiple up- and downward modulations (Figs. 1A, 2C, D, 3F). Cluster 4 (n = 75, N_infants/families_ = 2, N_adults_ = 2, Fig. 1A) included short syllables (17 ± 4 ms) of a high mean frequency (45.4 ± 4.1 kHz). Cluster 5 (n = 398, N_infants/families_ = 13, N_adults_ = 38, Figs. 1A, 2B–D, 3F) also comprised of short syllables (18 ± 10 ms), with them having a low mean frequency (16.0 ± 3.1 kHz). Finally, Cluster 6 (n = 33, N_infants/families_ = 3, N_adults_ = 22, Figs. 1A, 3F) contained long syllables (295 ± 92 ms) with the highest bandwidth (14.3 ± 5.7 kHz). Spectrograms of calling bouts from all four infant age classes can be seen in Fig. 2.

### Impact of age, context, and interaction partners on occurrence of clusters

To investigate whether next to age-dependent changes also the feature of the independence of social context (“vocal play”) is fulfilled, we used infant vocalizations recorded in two contexts. In the experimental isolation, infants were separated from their siblings and mother and recorded in an acoustic-damped room, while in the natural isolation infants were recorded in the nest boxes when the mother was absent. While during experimental isolation infants were alone, in natural isolation infants could be alone or together with up to two siblings. Per cluster we performed binomial Generalized Linear Mixed Models (GLMMs) using the cluster occurrence (yes/no) as test variable and infant age classes, context, and number of present infants as predictors while controlling for sender identity. The GLMMs were only calculated for Clusters 1–5, because Cluster 6 occurred predominantly in adults. For age class, significant effects on syllable occurrence were found for Clusters 1, 3, 4, and 5 (χ^2^ ≥ 8.23, df = 3, p ≤ 0.042; Tab. S2). Across infant age classes, the occurrence of Cluster 1 was decreasing with age, while the occurrences of Clusters 3 and 5 were increasing (Fig. 1B). The occurrence of Cluster 4 was higher in the first two infant age classes than in the latter two. For the predictor context, significant effects on syllable occurrence were only shown for Clusters 1 and 5 (χ^2^ ≥ 7.94, df = 1, p ≤ 0.005; Tab. S2). Cluster 1 was significantly more often produced in experimental isolation, while Cluster 5 was significantly more often emitted in natural isolation. The number of potential interaction partners (siblings) had no significant effect on syllable occurrence (χ^2^ ≤ 2.63, df = 2, p ≥ 0.268; Tab. S2).

### Syntactic structure of calling bouts

To investigate the sequential order of the syllable types, transition networks for each age class were calculated (Fig. 3, Tab. S3). Thereby, network density and clustering coefficient (i.e., network metrics describing the complexity of a network; the higher the more complex the networks) trended to increase with age, showing that the networks became more complex as the infants got older (network density: 0–1 pd: 0.100, 10–11 pd: 0.400, 17–18 pd: 0.300, 26–27 pd: 0.367; clustering coefficient: 0–1 pd: 0.000, 10–11 pd: 0.389, 17–18 pd: 0.528, 26–27 pd: 0.361). The maximum values were calculated for the adults (network density: 0.467; clustering coefficient: 0.633). Age class 0–1 pd showed the simplest structure since most of the syntax was based on repetitions of Cluster 1 (Fig. 3A). During age class 10–11 pd the structure became more complex: repetitions of single syllables were common (mainly Clusters 3, 4, and 5), and combinations of different syllable types were emitted in a random order (Fig. 3B). However, the appearance of a syntactic structure similar to that observed for the adults appeared by age class 17–18 pd (Fig. 3C, D). Thereby, the transition between Clusters 3 and 5, which is typical for the adult trill call (Fig. 3E), appeared from age class 10–11 pd, gradually becoming more evident in later infant age classes. In addition, self-loops (i.e., repetitions of the same syllable type) showed the characteristic repetitions of Clusters 3 and 5 of the adult trill call, which became more prominent with increasing age of the infants. In addition, at age class 26–27 pd self-loops of Cluster 1 decreased while the connection between Cluster 1 and Cluster 3 became more prominent, which can be seen more clearly in the transition networks without self-loops (Fig. S3).

**Figure 4:**
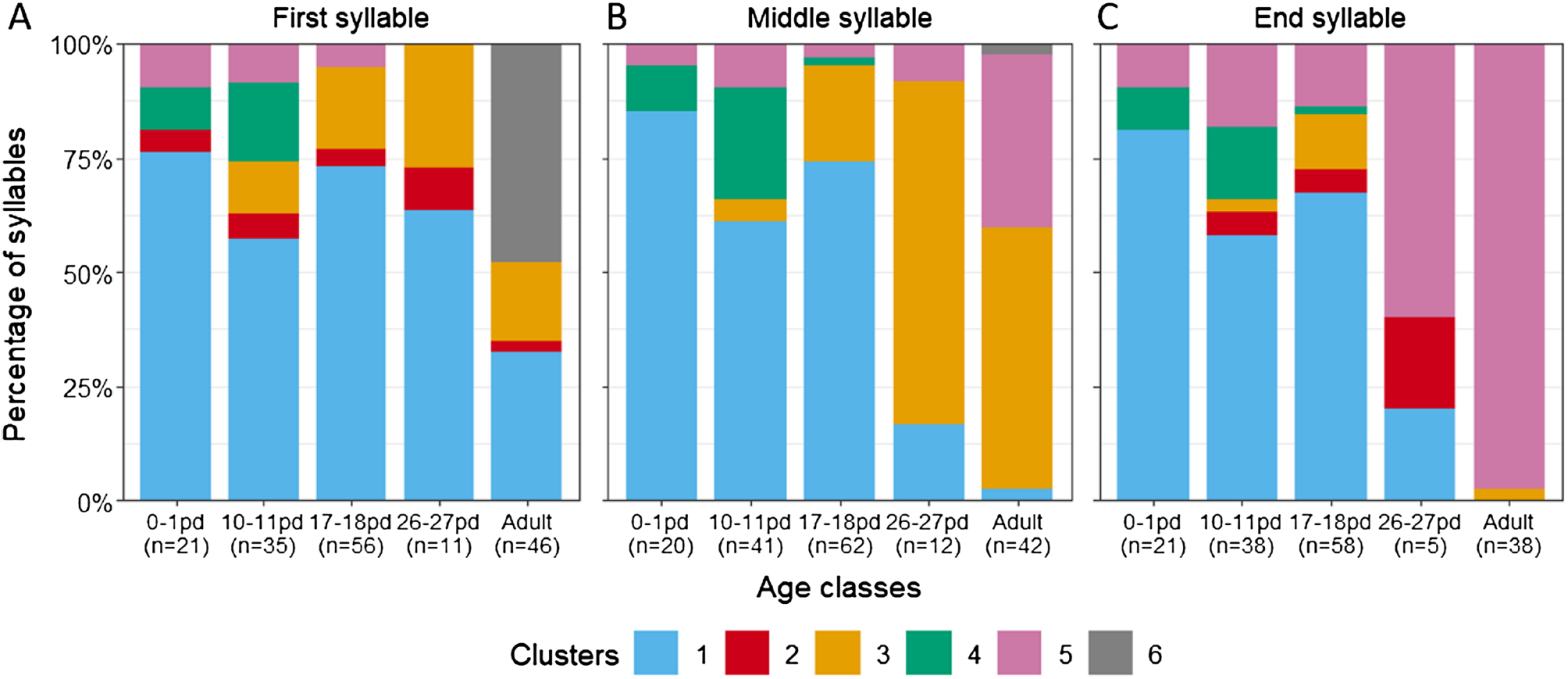
Distribution of clusters for the three structural parts of the adult trill call across age classes. (**A**) First syllable, (**B**) Middle syllable and (**C**) End syllable. pd = postnatal day; n = number of syllables.

To investigate the development of the three structural parts of the adult trill, we performed GLMMs to examine the impact of age class on the occurrence of different syllable types at the start, middle, and end of a calling bout. The first syllables of the calling bouts belonged mainly to Clusters 1, 3, and 6 (Fig. 4A). Compared to the adult age class, the occurrence of Cluster 1 was significantly higher in the age classes 0–1, 10–11 and 17–18 pd (lsmeans: Z ≥ 2.7, p ≤ 0.028) but not in the age class 26–27 pd (Z = 2.0, p = 0.174, Tab. S4). For Cluster 3 no effect of age was found while Cluster 6 was only documented as first syllable for the adult age class (Fig. 4A). The middle syllable of a calling bout belonged mainly to Clusters 1, 3, and 5 (Fig. 4B). For Cluster 1 infants showed a higher occurrence of syllables compared to the adults for age classes 0–1, 10–11, and 17–18 pd (Z ≥ 2.9, p ≤ 0.013) but not in the age class 26–27 pd (Z = 1.8, p = 0.250, Tab.S4). Clusters 3 and 5 significantly increased in age classes 10–11 and 17–18 pd (Z ≤ − 2 .51, p ≤ 0.047), whereas for age class 26–27 pd no difference to the adult age class was found (Z ≥ -2.27, p ≥ 0.090, Tab. S4). The end syllable of a calling bout belonged mainly to Clusters 1 and 5 (Fig. 4C). For Cluster 1, again a significant decrease in the syllable occurrence was found across the age classes (χ^2^ = 10.2, df = 4, p = 0.037; Tab. S4). However, the pairwise comparisons between the infant age classes and the adult age class were not significant. In contrast, for Cluster 5 the syllable occurrence was lower in age classes 0–1, 10–11 and 17–18 pd (Z ≤ -3.34, p ≤ 0.003) but not different in age class 26–27 pd (Z = − 2.2, p = 0.103, Tab. S4) compared to the adult age class.

### Temporal structure

**Figure 5:**
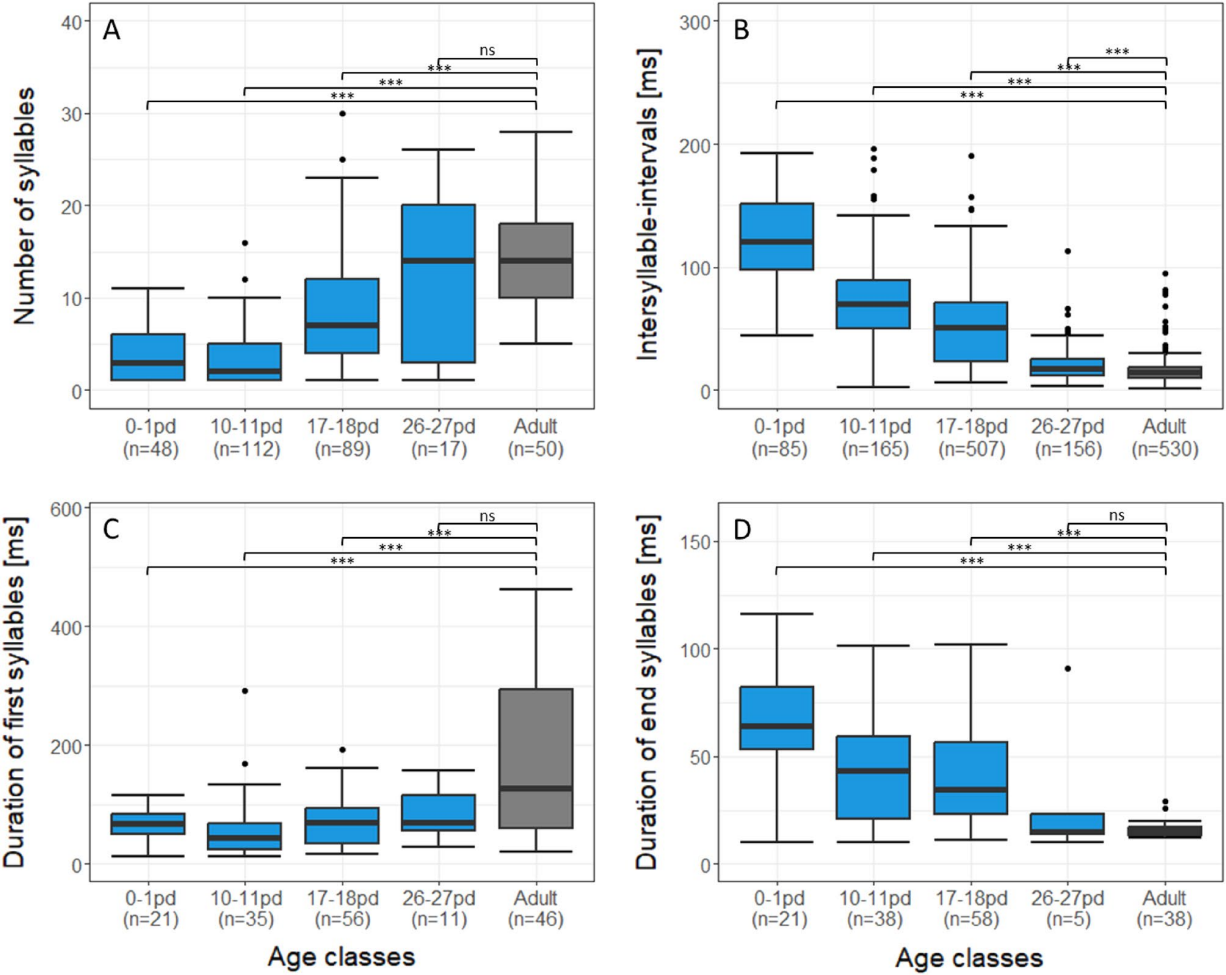
Temporal development of gray mouse lemur vocalizations. (**A**) Syllables per bout, (**B**) Intersyllable-interval, (**C**) Duration of first syllable and (**D**) Duration of end syllable. Boxplots represent median (black line), interquartile range (box) and non-outlier range (whiskers). n = number of syllables; Blue = infant age classes; Gray = adult age class; pd = postnatal day; ns = non-significant; *** p < 0.001.

To explore the development of the temporal structure in the calling bouts, we performed linear-mixed effects models (LMMs) to investigate the effect of age class for the syllables per bout, the intersyllable-interval, and the durations of the first, middle and end syllables (Tabs. S5, S6). All temporal parameters significantly differed between the age classes (χ^2^ ≥ 22.1, df = 4, p < 0.001, Tab. S6). Pairwise comparisons between the adult and infant age classes revealed that infants produced significantly less syllables per calling bout and shorter first syllables in age classes 0–1, 10–11 and 17–18 pd compared to the adult age class (t ≤ -3.98, p ≤ 0.001), while there was no difference between the adults and the age class 26–27 pd (t ≤ − 2.4, p ≥ 0.053, Fig. 5A, C, Tab. S6). In contrast, the duration of the middle and end syllables showed the reverse pattern, being longer in age classes 0–1, 10–11 and 17–18 pd compared to adults (t ≥ 3.75, p ≤ 0.001), with no difference between age class 26–27 pd and the adults (t ≤ 2.06, p ≥ 0.155; Fig. 5D, Tab. S6). On the other hand, the intersyllable-intervals continuously decreased across age classes, with all infant age classes differing significantly from the adults (t ≥ 6.31, p < 0.001, Fig. 5B, Tab. S6).

### Acoustic structure of syllable types

**Figure 6:**
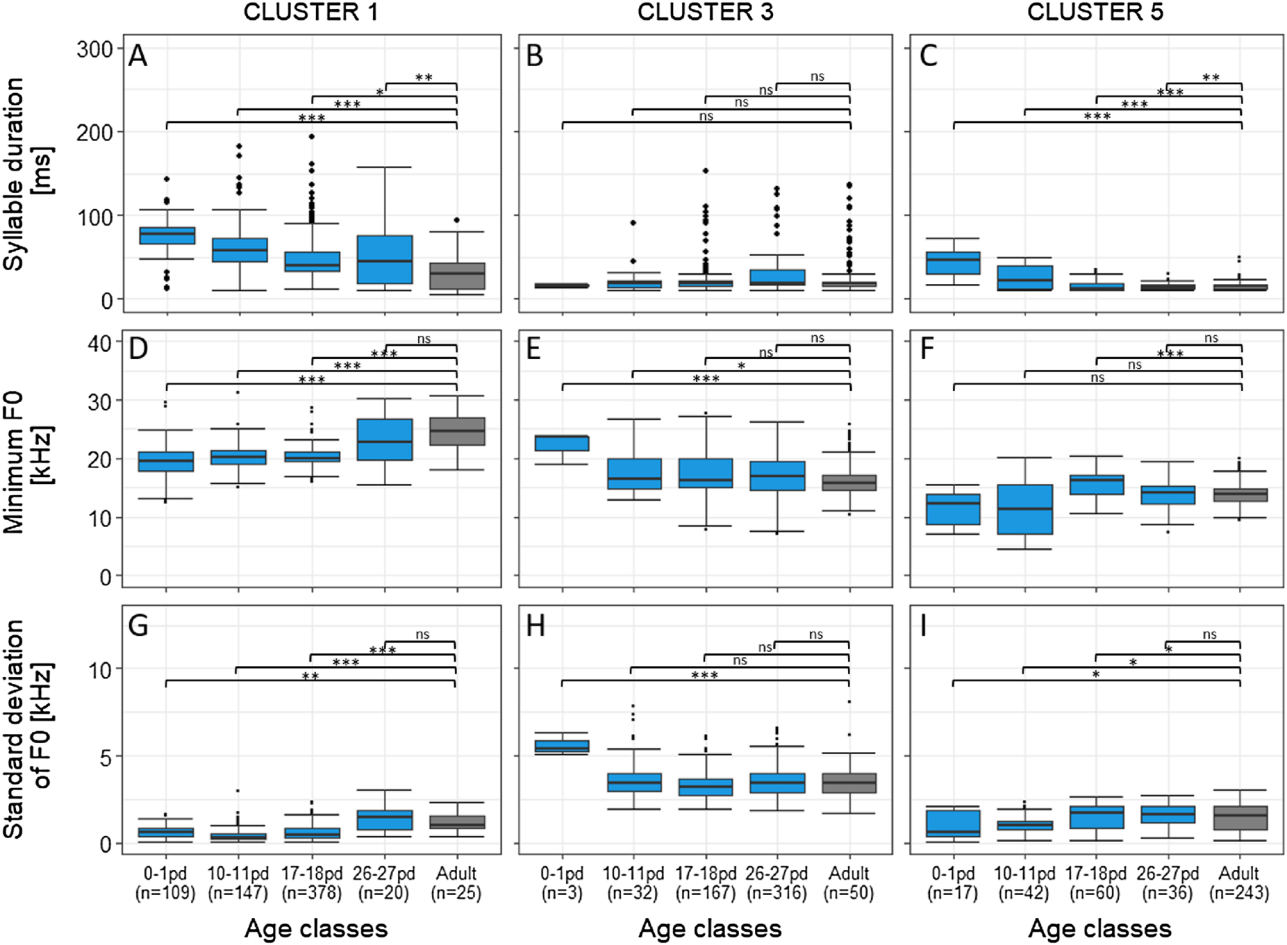
Development of acoustic structure of gray mouse lemur vocalizations. (**A**–**C**) Syllable duration of Clusters 1, 3, and 5, (**D**–**F**) Minimum fundamental frequency of Clusters 1, 3, and 5, (**G**–**I**) Standard deviation of fundamental frequency of Clusters 1, 3, and 5. Boxplots represent median (black line), interquartile range (box) and non-outlier range (whiskers). pd = postnatal day; n = number of syllables; F0 = fundamental frequency; Blue = infant age classes; Gray = adult age class; ns = non-significant; ***p < 0.001, **p < 0.01, *p < 0.05.

To explore age-dependent changes in the tempo-spectral structure of syllable types, we performed LMMs to investigate the impact of age class on both temporal and spectral parameters (Tabs. S7–S10). We focused our analyses on Clusters 1, 3, and 5 because only for these clusters sufficient syllables occurred in all age classes. In contrast to our expectation, the tempo-spectral changes showed no consistent results. For all temporal parameters, a decrease across age classes was found for Clusters 1 and 5 (χ^2^ ≥ 12.05, df = 4, p ≤ 0.020), whereas Cluster 3 showed no significant differences across age classes (χ^2^ ≤ 6.95, df = 4, p ≥ 0.139, Fig. 6A–C, Tab. S10). For the spectral parameters, we obtained opposite results for Clusters 1 and 5 compared to Cluster 3. Specifically, for Clusters 1 and 5 fundamental frequency and frequency modulation parameters increased (χ^2^ ≥ 11.36, df = 4, p ≤ 0.023; except bandwidth for Cluster 5). In contrast, for Cluster 3 the same parameters decreased (χ^2^ ≥ 23.84, df = 4, p < 0.001, Fig. 6D–I, Tab. S10).

## Discussion

Our study shows that gray mouse lemur infants produce variable vocal streams of different syllable types in isolation contexts independent of the presence of a vocal referent. Thereby, the syntactic and temporal structure of the infant calling bouts gradually resembles the adult trill call by adapting the sequential order of the syllable types, the length of the calling bouts, the intersyllable-intervals and the duration of the middle and end syllables. The syllable type and duration of the first syllable were more variable than for the rest of the calling bouts, indicating the potential to encode individuality of the sender. Age-dependent changes in the acoustic parameters of the vocalizations were inconsistent across syllable positions and clusters, which we would expect to be uniform if ontogenetic changes were a result of physical maturation of the vocal production apparatus. Therefore, our findings suggest that the development of adult trill calls during infancy by increasing frequency modulation of syllables and by combining specific syllable types into a complex sequence cannot solely be explained by physical maturation.

In the following we will discuss to which extent gray mouse lemur infants exhibit the features of animal babbling proposed by Elowson et al.^10^ and Fernandez et al.^11^ during the acquisition of the adult trill call (see for summary Tab. S11). Several gray mouse lemur infants housed in different housing rooms produced almost all syllable types (except Cluster 6) in all infant age classes, thus fulfilling the feature of *Universality*. Infants produced no trill calls at birth but a subset of adult syllable types, thus the feature of *Syllable subset acquisition* is fulfilled. The occurrence of the different syllable types was less affected by context and infants produced all clusters irrespective of whether they were alone or siblings were present (“vocal play”). This supports the feature of *Independence of the social context* similar to human infants, bats, and marmosets (e.g.,^1,10,11^). The syllable types were produced in bouts showing repetitions of the same syllable type (self-loops), fulfilling the feature of *Rhythmic and Repetitive*. Thereby, our results demonstrate that the occurrence of the complex, frequency modulated syllables increased with age and the syllable sequences showed the typical repetitions and connections of specific syllable types characteristic for the adult trill calls, corresponding to findings in bats and marmosets (e.g.,^10–12^). In detail, at the first infant age class, calling bouts were characterized by repetitions of the “simple” syllable type of Cluster 1 with almost no frequency modulation. As the infants matured, the occurrence of Cluster 1 decreased, while the occurrence of frequency modulated syllables increased. At the age class 10–11 pd, connections between different syllable types occurred, which were at some degree randomly associated. At the age classes 17–18 and 26–27 pd the combination of syllable types became more structured, gradually resembling the adult trill call. Thereby, the characteristic sequential order of the adult trill call (self-loops of Clusters 3 and 5, connections between Clusters 3 and 5) became progressively more prominent as the infants got older. Cluster 3 became the most prominent cluster for the middle part of the trill, whereas Cluster 5 almost always served as the end part. By combining syllable types into sequences typical for adult trills, the feature of *Babbling bout composition* into adult sequences is fulfilled. Finally, Scheumann et al.^30^ reported that gray mouse lemur infants produce vocal streams of high vocal plasticity during vocal exchanges with their mother, therefore fulfilling the feature of *Facilitating caregiver interactions*.

However, our study provides only weak evidence that the babbling feature of a *Peak of high vocal plasticity* during infancy is fulfilled. During infancy, an expansion of two additional syllable types and the highest score for network density occurred already at age class 10–11 pd. This might be an indication that at least a phase of more variable combinations is exhibited during the age class 10–11 pd. However, the expansion of two syllable types was only minor and network density was on a similar level as for the adults. This might indicate a prolonged vocal plasticity due to brain reorganization similar to marmosets^21^. Furthermore, we have to admit that the K-means clustering as a hard clustering algorithm might merge syllable types to larger categories (especially when syllable types are underrepresented in the data set), which can result in a lower number of acquired syllable types. Nevertheless, we opted for the cluster analysis instead of visual classification to avoid a human observer bias and an inflation of syllable types due to minor changes in the contour of the fundamental frequency.

Concerning the development of the first syllable of the adult trill, while in the adults Cluster 6 predominated as a first syllable, in the infant age classes mostly Cluster 1 and Cluster 3 were used as a first syllable. An explanation for this discrepancy could be, that Cluster 6 is the result of a mergence of different types of syllables. Particularly, flat syllables (e.g., Cluster 1, Cluster 2) might merge during ontogeny with modulated syllables (e.g., Cluster 3) resulting in the syllable type of Cluster 6. This is reflected in the contour of Cluster 6 (Fig. 1A), as it almost always starts with a whistle part (similar to the contour of syllables of Cluster 1 and Cluster 2) turning over in a modulation part (similar to the contour of syllables of Cluster 3)^43^. Thus, with increasing age syllable merging may result in the long and highly modulated syllables of Cluster 6, similar to findings in bats^44,45^. In addition, comparable to previous studies on individual vocal signatures showing a high level of individual distinctiveness at the initial modulation of the trill^39,43^, the first syllable of the adult trill was more variable than the middle and end syllables. Thus, the structure of the first syllable may undergo changes even after the weaning age. Therefore, after developing the trill as a call type, an additional individualized phase of vocal plasticity (= individual syllable combination) may take place, which we unfortunately cannot test with our data. Furthermore, age-dependent occurrence of syllable types might also be related to the behavioral response of the parents, which the calls elicit. The higher occurrence of Cluster 1 in the experimental isolation and at the younger age classes indicates a higher biological relevance of this cluster to elicit the attention of the mother^34^. Thus, the decreased occurrence of Cluster 1 is related to the increased independency of the infants, similar to the decrease of twitter calls in marmosets^21^. Although, the contexts were not equally distributed across age classes (Tab. S12), the decrease of Cluster 1 was shown from age classes 10–11 to 17–18 pd where both contexts were sufficiently represented. It could be assumed that when infants start to become independent the need of a fast response by the mother is decreasing whereas the pressure to develop an individual signature for the coordination of social interactions with sleeping group members or mates increases^46,47^, favoring continued vocal plasticity of trills after weaning. Thus, longitudinal studies recording infants from birth until social and sexual maturation are necessary.

Of high interest are the inconsistent age dependent changes in the acoustic parameters of the syllables. Age dependent changes in the acoustic parameters of calling bouts and syllables are discussed to be related to physical maturation of the vocal production system as the infants grow^21^. Thus, the increase of the number of syllables in a calling bout and the decrease in the intersyllable-interval can be explained by an increase in lung capacity, enabling the infants to produce longer calling sequences but also longer syllables^21^. Thus, we would expect an increase in syllable duration within the calling bout (first, middle, end) as well as for duration of specific syllable types. However, we found mixed results for both the syllable position and the syllable types. Whereas the duration of the first syllable increased across age classes, the duration of the middle and end syllables decreased. We further found inconsistent patterns in age-dependent changes of the fundamental frequency. It was expected that an increased body size would result in a lower fundamental frequency^21,48^. We found this pattern only for Cluster 3 whereas for Clusters 1 and 5 the opposite was observed. The increase of fundamental frequency with age is consistent with other species producing ultrasonic calls and indicates that the inverse relationship between fundamental frequency and body size found for mammals communicating in the audible range may not account for animals producing vocalizations in the ultrasonic range^49^. However, since both clusters are tonal calls, we would assume that in non-human primates they are based on the same vocal production mechanism^50^. The inconsistency in age dependent changes based on syllable position and syllable type makes it unlikely that this can solely be an effect of physical maturation of the vocal apparatus or internal auditory feedback. This is in accordance with findings in marmosets where acoustic changes could not fully be explained by physical growth but showed evidence for social-reinforced vocal learning^18,29,51^. Since vocal exchanges between gray mouse lemurs mothers and infants have been observed^30^, further research investigating the impact of these social interactions on the development of the trill call might help to detangle the effect of physical maturation and social learning.

For human infants external auditory feedback is a prerequisite for the transition from pre-canonical to the canonical babbling stage^8,9^. In the past, isolation and cross-fostering experiments were conducted in primates, arguing that primate vocalizations are innate since without external input the species-specific vocalizations are developed (review:^28^). Therefore, even though features of animal babbling are present in gray mouse lemurs, it does not necessarily imply that vocal learning as in human babbling takes place. Today, isolation experiments are no longer allowed due to ethical and legal restrictions. Thus, the case study of Kuhn^33^ in 1989 investigating two gray mouse lemur infants (one male, one female) isolated from their mother on the second day of life and reared by hand until weaning is of scientific value. The socially deprived male infant produced the adult trill call with an ontogenetic delay of 10 days compared to the mother-reared brother. Kuhn^33^ described the trill of the socially deprived male showing a high variability in the frequency contour and a double initial modulation (i.e., duplication of the typical initial modulation of adult trills), which was not documented in the other 21 mother-reared infants of the study. Interestingly, the delayed pattern is similar to that of deaf human infants, where the onset of the pre-canonical babbling is delayed and the contour of the uttered syllables deviates from those of the hearing infants^6^. After weaning, the socially-deprived infants were reunited with mother-reared conspecifics and the socially-deprived male developed a trill pattern which was similar to mother-reared conspecifics^31^. The socially deprived female did not produce a trill during the study and, unfortunately, we have no information on whether she produced trills in her later life. This indicates that social input even at a later developmental stage can shape this general trill template to a certain extent and is in accordance with a study of Hafen et al.^31^, who showed vocal convergence between adult male gray mouse lemurs independent of morphology or genetics.

All in all, in this study we demonstrated that gray mouse lemur infants show some features of animal babbling during the development of the adult call type trill, namely *Universality, Syllable subset acquisition*, *Independence of social contexts*, *Rhythmic and Repetitive* and *Babbling bout composition*. We found no strong support for a *Peak of vocal plasticity*. Instead, gray mouse lemur infants increased the occurrence of more complex syllable types with age, gradually developing a sequential order resembling the adult trill. Further studies have to investigate to which extent the development of the adult trill call can be modulated by auditory-feedback or socially-reinforced vocal learning. Thereby, it would be interesting whether vocal plasticity is restricted to infancy or occurs also during adulthood to adapt to the environment and social partners.

## Materials and methods

### Animal ethics

Infant and adult vocalizations originated from the sound archive of the Institute of Zoology, University of Veterinary Medicine Hannover, Germany. The recordings were conducted with animals of the captive self-sustaining gray mouse lemur colony of the institute between 2003 and 2012. The housing conditions of the breeding colony were regularly licensed and proved by the local veterinary authorities (license no.: 42502/1TiHo). Depending on the respective legislation for the year of the recordings, the studies were performed in accordance with the law of the European Community regulations about the protection of experimental animals and the guidelines of the German Animal Welfare Act and approved by the Niedersächsisches Landesamt für Verbraucherschutz und Lebensmittelsicherheit, Germany (license no.: 33.9-42502-05-11A117; 33. 12-42502-04-14/1454). Our study is reported in accordance with all applicable items of the ARRIVE Guidelines.

### Data sets and preparation

Infant calling bouts were either recorded in the animal housing room within the nest boxes of a mother with her offspring or during experimental paradigms in a sound-damped chamber^30,34^ using recording equipment for ultrasonic frequencies (Tab. S13). Since mouse lemurs utter vocalizations outside the human hearing range, it was not possible by the observer to differentiate between infant and mother vocalizations. To exclude a potential contamination with mother calls, high frequency infant calling bouts were only taken from vocal recordings when the mother was not present (mother was outside the nest box for at least 3 min^30^), isolation or playback experiments with infants, or strong manipulation of the infants^34^. A calling bout was defined as a sequence of syllables with intersyllable-intervals of less than 200 ms^34^. A syllable was defined as the smallest vocal unit of continuous sound energy. For infants, a total of 358 calling bouts resulting in 2129 syllables from 14 different families (recorded in the animal housing room) and 13 infants from experimental paradigms were used. To represent different developmental stages, infant calling bouts of four different age classes were selected: (a) 0–1 pd after birth reflecting vocal behavior soon after birth (204 syllables in 56 calling bouts), (b) 10–11 pd after birth reflecting the nursing period (462 syllables in 140 calling bouts), (c) 17–18 pd after birth representing the time infants start leaving the nests on their own (1250 syllables in 144 callings bouts) and (d) 26–27 pd after birth representing weaning^33^ (213 syllables in 18 calling bouts).

For adult vocalizations, the data set for trills from Romero-Mujalli et al.^37^ was used, which comprised of 50 trills (= calling bouts; n = 722 syllables) from 38 mouse lemurs recorded during social-encounter experiments.

### Acoustic measurements

For the acoustic analysis the software Praat (Version 5.4.04, Phonetic Sciences, University of Amsterdam, Netherlands^52^) and the GSU Praat Tools 1.9 scripts^53^ were used. To characterize the acoustic structure of each syllable, a custom-written script was used to measure three temporal, six spectral and one tonality-related parameters (Table 1).

**Table 1:** Description of measured acoustic parameters.

Parameter	Description
**Temporal parameters**	
Syllable duration [ms]	Time between the onset and the offset of a syllable
Time of minimum F0 [ms]	Time between the onset and the minimum of the fundamental frequency
Time of maximum F0 [ms]	Time between the onset and the maximum of the fundamental frequency
**Spectral parameters**	
Minimum F0 [kHz]	Minimum fundamental frequency of a syllable
Maximum F0 [kHz]	Maximum fundamental frequency of a syllable
Mean F0 [kHz]	Mean fundamental frequency of a syllable
Standard deviation of F0 [kHz]	Standard deviation of the fundamental frequency of a syllable
Bandwidth [kHz]	Difference between maximum and minimum frequency of a syllable
Mean slope [kHz/s]	Mean absolute slope of the fundamental frequency
**Tonality-related parameters**	
Voiced percentage [%]	Ratio of voiced frames to the sum of frames of a syllable

### Unsupervised cluster analysis

An unsupervised clustering model (K-means) was used to define distinctive syllable types, as k-means cluster analysis is an unbiased, simple and at the same time computationally efficient algorithm, which uses quantitative data and makes very few assumptions for the categorization task^37,54,55^. Syllables of bad quality (e.g., overlaid or silent syllables) were excluded from the analysis. Therefore, the unsupervised cluster analysis run with 1855 syllables (0–1 pd: 141 syllables, 48 calling bouts; 10–11 pd: 294 syllables, 112 calling bouts; 17–18 pd: 623 syllables, 89 calling bouts; 26–27 pd: 180 syllables, 17 calling bouts; adult: 617 syllables, 50 calling bouts). Unsupervised cluster analysis was performed, as it enabled the automation of the categorization task and to avoid subjectivity introduced by human observers. Prior to the use of the model, the independence of the acoustic parameters was tested by calculating pairwise Pearson correlations between them. For acoustic parameters with a correlation coefficient higher than 0.7 only one of them was included in the final data set (Fig. S1). The data was beforehand standardized (z-score), to ensure that the different range of the units of measurements did not falsify the analysis. The K-means model was iteratively run (Settings: clusters = 100, repetitions = 500, random starts = 100). Then, an elbow figure was drawn considering the ratio between the total within-clusters Sum of Squared Errors (SSE) and the total SSE. The optimal number of clusters *k*^*opt*^ was automatically decided by the model as the intersection point *k* of two lines that best fit the elbow data^37,54^. Each syllable was then labelled with its corresponding cluster ID. The clusters were also visualized in a bi-dimensional representation obtained by a t-SNE analysis. All analyses were carried out in R^56^ (Version 4.0.5; 2021-03-31; accessed using the integrated development environment RStudio^57^ (Version 1.4.1106); packages: t-SNE—“Rtsne” (Version 0.15); K-means model—“cluster” (Version 2.1.1); Elbow function based on Romero-Mujalli et al.^37^ (github R package: https://github.com/danielrm84/tihoCluster); graphical illustrations—“ggplot2” (Version 3.3.2)).

### Impact of age, context, and interaction partners on occurrence of clusters

To investigate how infant age classes, context, and presence of interaction partners affected the occurrence of the individual clusters, binomial Generalized Linear Mixed Models (GLMMs) were calculated for each cluster (glmer—“lme4” (Version 1.1-27.1)) using Cluster occurrence (yes/no) as test variable and infant age classes, context, and number of present infants as predictors while controlling for sender identity. For each model ANOVA tables with F-tests and p-values were calculated (Anova—“car” (Version 3.1-0); Tab. S2). In case a parameter had a significant effect on the occurrence of an individual cluster, pairwise comparisons were carried out between the different categories of the parameter using sidak correction to control for multiple testing (lsmeans—“emmeans” (Version 1.6.3)).

### Syntax analysis and transition networks

Based on the results of the unsupervised cluster analysis, transition matrices were calculated (one transition matrix per age class) to investigate whether the sequential order of the syllables differed between age classes. Thus, for each calling bout, transitions were counted for pairs of consecutive syllables. As some syllables were excluded from the cluster analysis due to their bad quality, transitions were calculated only for sequences where the consecutive syllables were included in the cluster analysis (Number of available transitions: 0–1 pd = 85, 10–11 pd = 166, 17–18 pd = 506, 26–27 pd = 156, Adults = 531). The frequency at which each transition occurred was divided with the total sum of transitions for the corresponding age class, thus acquiring transition matrices. Finally, this information was used to plot the transition matrices in the form of network diagrams. To make the networks comparable across age classes, the width of the nodes and the edges was proportional to the occurrence of the corresponding cluster or transition respectively for each age class. The thickness of edges corresponded to the values of the transition matrices. The size of the nodes was calculated based on each cluster occurrence in the respective age class, with the occurrence divided by the total number of transitions per age class. The network diagrams were illustrated in Cytoscape^58^ (Version 3.9.1). In addition, the network density and the clustering coefficients for each age class were calculated in Cytoscape. Network density describes the number of edges in a transition network as a proportion of the total count possible. Clustering coefficient on the other hand refers to the mean of the ratios for each node of the number of edges between its neighbors and the maximum number of edges that could possibly exist.

Since adult trill calls compose of three distinguishable parts^31,41^, for calling bouts consisting of at least three syllables it was noted in which cluster the first, the middle and the end syllable were clustered to. The first and end syllables reflected the beginning and end of the calling bout. For the middle syllable we divided the number of total syllables by 2 and took the respective syllable. In the case of non–whole numbers (e.g., 5.5) the result was rounded up (e.g., 6). To evaluate how the syllables differed between the age classes, GLMMs were calculated for each syllable position and each cluster. Thus, the occurrence of Cluster (yes/no) was used as test variable and age class as predictor while controlling for sender identity. If the ANOVA of the GLMM was significant, pairwise comparisons between the adult age class and the infant age classes were conducted using sidak correction to control for multiple testing.

### Age dependent changes in the acoustic structure

To investigate how the temporal parameters of the calling bouts differed between the age classes, the effect of age on the bout length (= number of syllables per bout), the intersyllable-interval (= duration from offset of the first syllable to the onset of the following syllable) and the duration of the first, middle and end syllables were calculated using linear mixed-effects models (lmer—“lme4” (Version 1.1-27.1)). Additionally, age-dependent changes in the acoustic structure were investigated for Clusters 1, 3, and 5. The acoustic parameter was used as test variable, age class as predictor variable and sender identity as random factor. If the ANOVA of the model yielded a significant difference between age classes, we conducted pairwise comparisons between the adult and infant age classes using the sidak correction to control for multiple testing.

### Supplementary Information

Supplementary Information.

## Data Availability

The raw dataset used for the manuscript is available in Zenodo repository at 10.5281/zenodo.10194218. Video and audio files are stored in the sound archive of the Institute of Zoology, University of Veterinary Medicine Hannover and are available on reasonable request from Dr. Marina Scheumann.
