# Supplementary material for: Features of animal babbling in the vocal ontogeny of the gray mouse lemur (Microcebus murinus)
Source: Sci Rep. 2023 Dec 4;13:21384. doi: 10.1038/s41598-023-47919-7 (PMC10696017; doi:10.1038/s41598-023-47919-7)
Supplement: Supplementary file 1 — Supplementary Information. [file 41598_2023_47919_MOESM1_ESM.docx]

Supplementary Materials for

**Features of animal babbling in the vocal ontogeny of the gray mouse lemur (*Microcebus murinus)***

Langehennig-Peristenidou et al.

*Corresponding author. Email: alexandra.langehennig-peristenidou@tiho-hannover.de

T**his PDF file includes:**

Fig. S1 to S3

Tab. S1 to S13

Fig.S1.

Correlation plots and Pearson correlation coefficients between the acoustic parameters. On the upper panel the correlation matrices are illustrated, while the Pearson correlation coefficients are shown on the lower panel. Parameters: duration = syllable duration, voiced = voiced percentage, minF0 = minimum F0, timeminF0 = time of minimum F0, maxF0 = maximum F0, timemaxF0 = time of maximum F0, meanF0 = mean F0, sDF0 = standard deviation of F0, yellow box: R > 0.7.


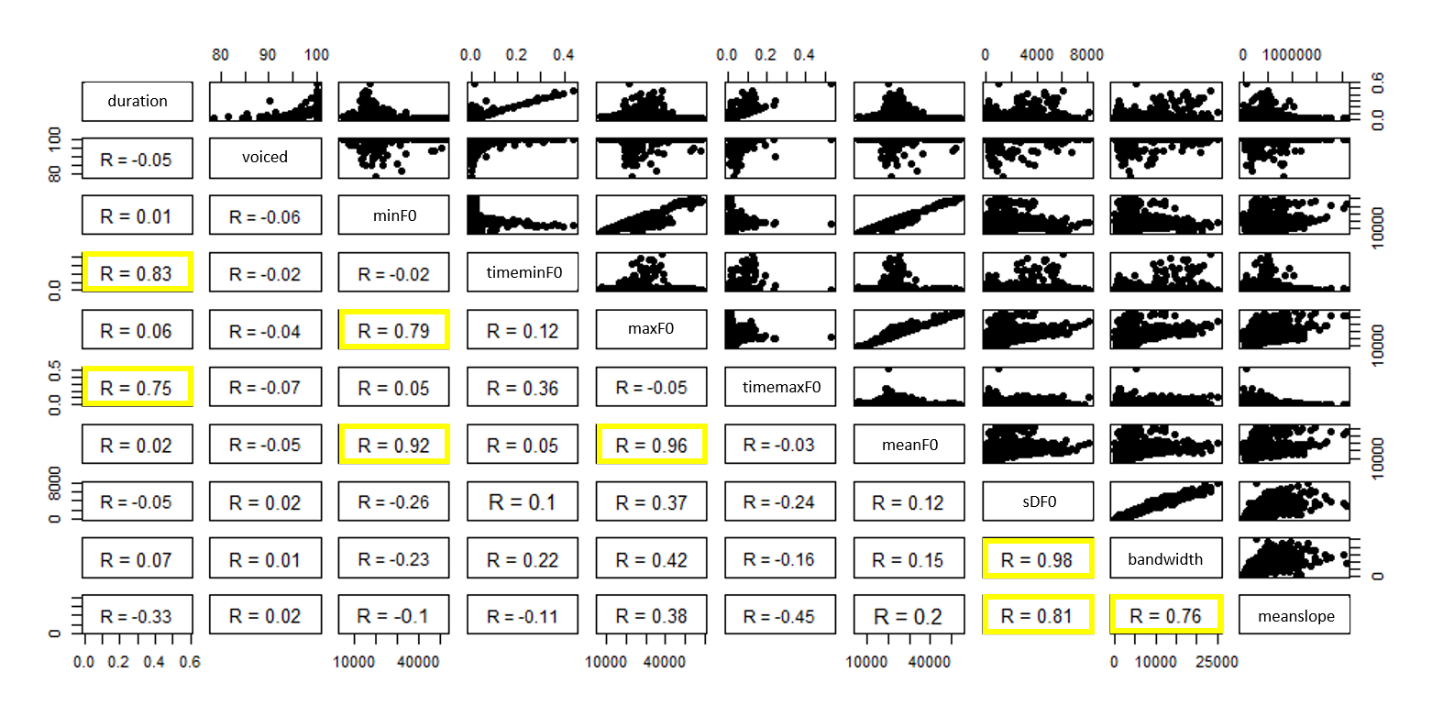


Fig.S2.

Plot showing the optimal number of clusters based on the Elbow method (unsupervised cluster analysis; K-means model). The model automatically decides the optimal number of clusters*^37,54^*.


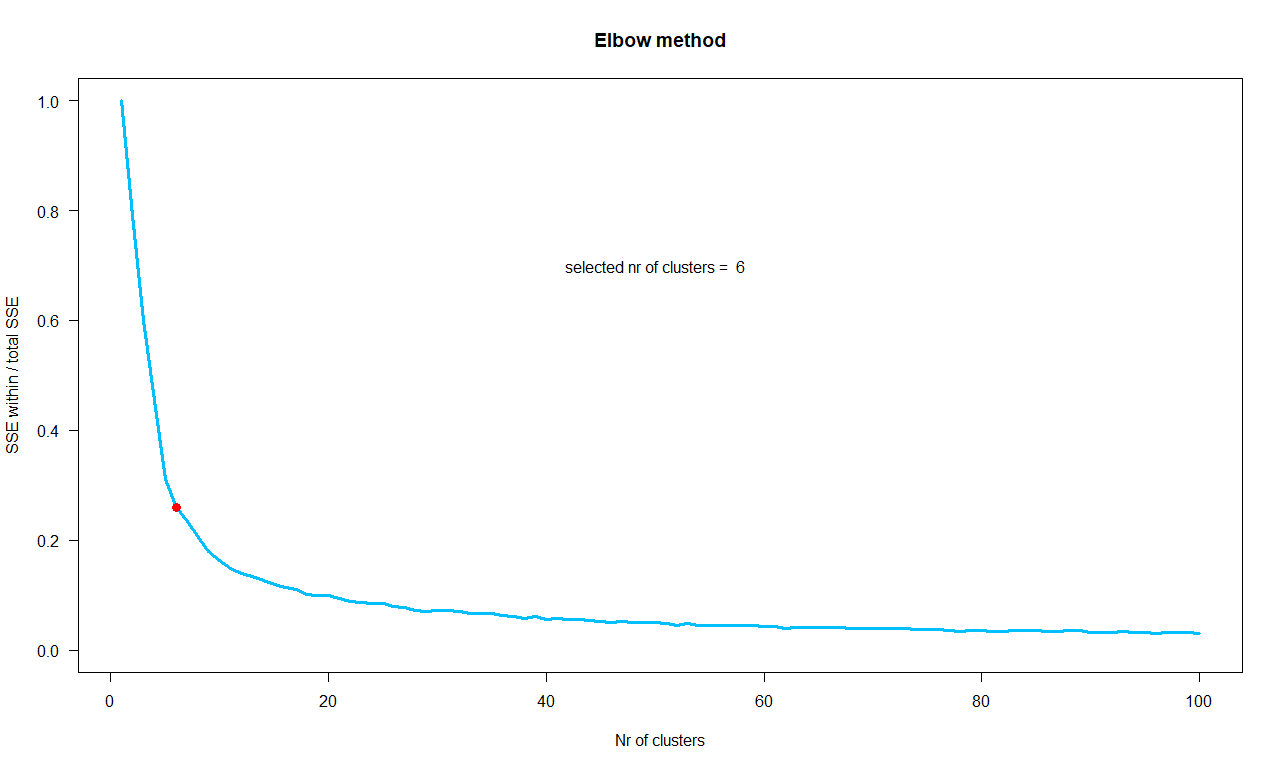


Fig.S3.

**Syntactic structure per age class without self-loops** (**A**) 0-1 postnatal days (pd), (**B**) 10-11 pd, (**C**) 17-18 pd, (**D**) 26-27 pd, (**E**) Adults with (**F**) a spectrogram of an adult trill call. Networks: The edges are directional, being colored in the same color as the previous syllable of a transition. The width of the nodes and the edges are proportional to the percentage of the transition in the respective age class. Spectrogram: Dash line marks the start of the syllable. Colored rectangle on the top marks the assigned cluster. pd=postnatal day.


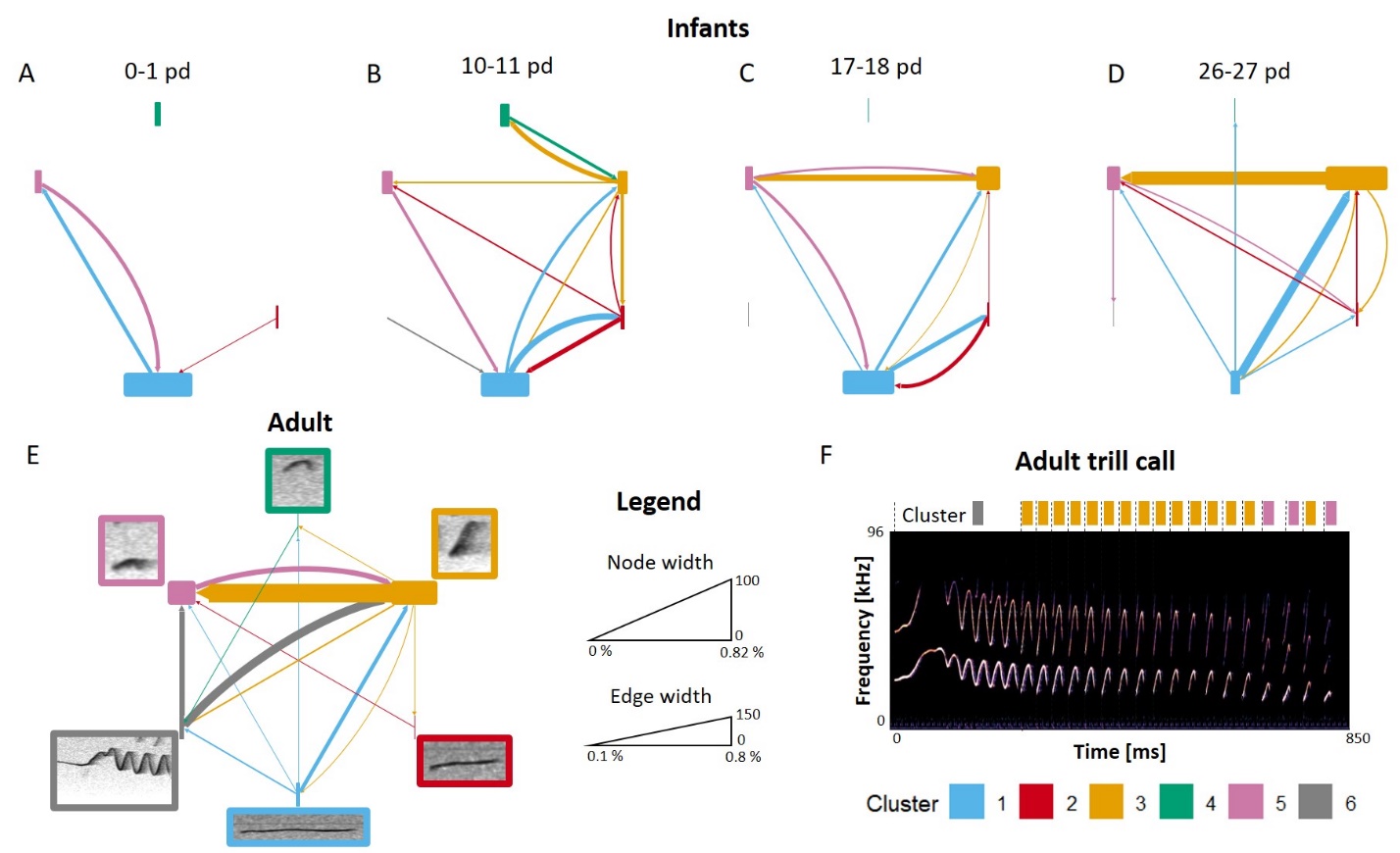


Tab.S1.

Acoustic description (mean ± standard deviation) of the clusters obtained by the K-mean clustering

algorithm. n = number of syllables.

|  | **Cluster 1 (n=679)** | **Cluster 2 (n=36)** | **Cluster 3 (n=634)** | **Cluster 4 (n=75)** | **Cluster 5 (n=398)** | **Cluster 6 (n=33)** |
| --- | --- | --- | --- | --- | --- | --- |
| **Temporal parameters** | | | | | | |
| Syllable duration [ms] | 55.80  ± 28.27 | 49.47  ± 48.60 | 23.70  ± 19.29 | 16.51  ± 4.12 | 17.96  ± 10.01 | 294.73  ± 92.45 |
| Time of minimum F0 [ms] | 18.21  ± 24.31 | 12.06  ± 16.12 | 6.44  ± 14.94 | 8.88  ± 6.88 | 3.48  ± 4.98 | 237.18  ± 109.81 |
| Time of maximum F0 [ms] | 41.60  ± 27.09 | 37.50  ± 40.77 | 15.77  ± 10.98 | 8.33  ± 3.27 | 12.97  ± 9.60 | 105.73  ± 90.89 |
| **Spectral parameters** | | | | | | |
| Minimum F0 [kHz] | 20.37  ± 2.38 | 19.64  ± 4.87 | 16.63  ± 3.26 | 43.11  ± 3.79 | 13.87  ± 2.76 | 15.92  ± 2.07 |
| Maximum F0 [kHz] | 22.63  ± 2.83 | 24.73  ± 4.94 | 26.58  ± 3.48 | 47.66  ± 4.82 | 17.85  ± 3.55 | 30.19  ± 5.13 |
| Mean F0 [kHz] | 21.39  ± 2.54 | 22.05  ± 4.30 | 22.31  ± 3.12 | 45.43  ± 4.13 | 16.04  ± 3.12 | 23.45  ± 2.93 |
| Standard deviation of F0 [kHz] | 0.62  ± 0.46 | 1.65  ± 1.58 | 3.47  ± 0.89 | 1.39  ± 0.82 | 1.41  ± 0.74 | 3.76  ± 1.60 |
| Bandwidth [kHz] | 2.26  ± 1.52 | 5.09  ± 4.39 | 9.95  ± 2.80 | 4.55  ± 2.96 | 3.97  ± 2.00 | 14.27  ± 5.65 |
| Mean slope [kHz/s] | 125.90  ± 123.32 | 391.93  ± 294.08 | 783.64  ± 193.87 | 648.12  ± 395.70 | 426.42  ± 252.07 | 463.35  ± 212.13 |
| **Tonality-related parameters** | | | | | | |
| Voiced percentage [%] | 99.90  ± 0.58 | 90.56  ± 4.08 | 99.97  ± 0.27 | 99.77  ± 1.13 | 100.00  ± 0.00 | 99.80  ± 0.68 |

Tab.S2.

Results of the GLMMs testing effects of infant age class, context and number of present infants on the occurrence of the respective cluster. Bold: p <0.05.

|  | Infant age classes  (df = 3) | | Context  (df = 1) | | Infants present  (df = 2) | |
| --- | --- | --- | --- | --- | --- | --- |
|  | χ^2^ | P - value | χ^2^ | P - value | χ^2^ | P - value |
| Cluster 1 | 91.71 | **<0.001** | 29.39 | **<0.001** | 0.21 | 0.902 |
| Cluster 2 | 3.14 | 0.371 | 0.88 | 0.349 | 0.64 | 0.726 |
| Cluster 3 | 128.85 | **<0.001** | 0.00 | 0.979 | 0.29 | 0.866 |
| Cluster 4 | 25.73 | **<0.001** | 0.00 | 1.000 | 2.63 | 0.268 |
| Cluster 5 | 8.23 | **0.042** | 7.94 | **0.005** | 1.27 | 0.530 |
| Cluster 6 | Not calculated because of low occurrence in infants | | | | | |

Tab.S3.

Weighted transition matrices and standardized node size used for network analyses. Transition matrices: Occurrences of each transition were divided by the corresponding number of transitions between the clusters of the corresponding age class. Standardized node size: Occurrence of the respective cluster divided by the number of transitions and multiplied by 1000. pd = postnatal day; n = total number of transitions.

| **Age class** | **Weighted transition matrices** | | | | | | | **Standardized**  **node size** |
| --- | --- | --- | --- | --- | --- | --- | --- | --- |
|  | Cluster | 1 | 2 | 3 | 4 | 5 | 6 |  |
| **0-1 pd**  **(n=85)** | 1 | 0.800 | 0.000 | 0.000 | 0.000 | 0.024 | 0.000 | 824 |
|  | 2 | 0.012 | 0.012 | 0.000 | 0.000 | 0.000 | 0.000 | 24 |
|  | 3 | 0.000 | 0.000 | 0.000 | 0.000 | 0.000 | 0.000 | 0 |
|  | 4 | 0.000 | 0.000 | 0.000 | 0.071 | 0.000 | 0.000 | 71 |
|  | 5 | 0.024 | 0.000 | 0.000 | 0.000 | 0.059 | 0.000 | 82 |
|  | 6 | 0.000 | 0.000 | 0.000 | 0.000 | 0.000 | 0.000 | 0 |
| **10-11 pd**  **(n=166)** | 1 | 0.554 | 0.024 | 0.012 | 0.000 | 0.000 | 0.000 | 590 |
|  | 2 | 0.018 | 0.012 | 0.006 | 0.000 | 0.006 | 0.000 | 42 |
|  | 3 | 0.006 | 0.012 | 0.084 | 0.018 | 0.006 | 0.000 | 127 |
|  | 4 | 0.000 | 0.000 | 0.012 | 0.096 | 0.000 | 0.000 | 108 |
|  | 5 | 0.012 | 0.000 | 0.000 | 0.000 | 0.114 | 0.000 | 127 |
|  | 6 | 0.006 | 0.000 | 0.000 | 0.000 | 0.000 | 0.000 | 6 |
| **17-18 pd**  **(n=506)** | 1 | 0.569 | 0.020 | 0.014 | 0.000 | 0.008 | 0.000 | 611 |
|  | 2 | 0.016 | 0.000 | 0.002 | 0.000 | 0.000 | 0.000 | 18 |
|  | 3 | 0.004 | 0.000 | 0.255 | 0.000 | 0.026 | 0.000 | 285 |
|  | 4 | 0.000 | 0.000 | 0.000 | 0.000 | 0.000 | 0.000 | 0 |
|  | 5 | 0.014 | 0.000 | 0.010 | 0.000 | 0.063 | 0.000 | 87 |
|  | 6 | 0.000 | 0.000 | 0.000 | 0.000 | 0.000 | 0.000 | 0 |
| **26-27 pd**  **(n=156)** | 1 | 0.051 | 0.006 | 0.032 | 0.006 | 0.006 | 0.000 | 103 |
|  | 2 | 0.000 | 0.013 | 0.006 | 0.000 | 0.006 | 0.000 | 26 |
|  | 3 | 0.006 | 0.006 | 0.660 | 0.000 | 0.051 | 0.000 | 724 |
|  | 4 | 0.000 | 0.000 | 0.000 | 0.000 | 0.000 | 0.000 | 0 |
|  | 5 | 0.000 | 0.006 | 0.000 | 0.000 | 0.135 | 0.006 | 147 |
|  | 6 | 0.000 | 0.000 | 0.000 | 0.000 | 0.000 | 0.000 | 0 |
| **Adult**  **(n=531)** | 1 | 0.015 | 0.000 | 0.015 | 0.002 | 0.002 | 0.006 | 40 |
|  | 2 | 0.000 | 0.000 | 0.000 | 0.000 | 0.002 | 0.000 | 2 |
|  | 3 | 0.002 | 0.002 | 0.478 | 0.002 | 0.073 | 0.006 | 563 |
|  | 4 | 0.000 | 0.000 | 0.000 | 0.000 | 0.000 | 0.002 | 2 |
|  | 5 | 0.000 | 0.000 | 0.021 | 0.000 | 0.318 | 0.000 | 339 |
|  | 6 | 0.000 | 0.000 | 0.034 | 0.000 | 0.021 | 0.000 | 55 |

Tab.S4.

Pairwise comparisons between the adult and infant age classes for the clusters, which showed a global significant effect of age class on the occurrence of the respective cluster using GLMMs. Bold: p < 0.05; pd = postnatal day; SE = Standard error.

| Contrast | | | Estimate | SE | Z – ratio | P – value |
| --- | --- | --- | --- | --- | --- | --- |
| FIRST SYLLABLE | | | | | | |
| Cluster 1 (χ² = 15.10; df = 4; p = 0.005) | | | | | | |
| 0-1 pd | - | Adult | 3.29 | 0.95 | 3.45 | **0.002** |
| 10-11 pd | - | Adult | 2.19 | 0.81 | 2.70 | **0.028** |
| 17-18 pd | - | Adult | 2.53 | 0.71 | 3.58 | **0.001** |
| 26-27 pd | - | Adult | 1.92 | 0.97 | 1.99 | 0.174 |
| MIDDLE SYLLABLE | | | | | | |
| Cluster 1 (χ² = 19.03; df = 4; p = 0.001) | | | | | | |
| 0-1 pd | - | Adult | 12.56 | 3.44 | 3.65 | **0.001** |
| 10-11 pd | - | Adult | 9.64 | 3.27 | 2.95 | **0.013** |
| 17-18 pd | - | Adult | 9.55 | 3.25 | 2.94 | **0.013** |
| 26-27 pd | - | Adult | 6.70 | 3.69 | 1.82 | 0.250 |
| Cluster 3 (χ² = 26.29; df = 4; p < 0.001) | | | | | | |
| 0-1 pd | - | Adult | -57.10 | 106.78 | -0.53 | 0.973 |
| 10-11 pd | - | Adult | -20.70 | 4.46 | -4.64 | **<0.001** |
| 17-18 pd | - | Adult | -19.10 | 4.35 | -4.39 | **<0.001** |
| 26-27 pd | - | Adult | -25.10 | 11.08 | -2.27 | 0.090 |
| Cluster 5 (χ² = 15.82; df = 4; p = 0.003) | | | | | | |
| 0-1 pd | - | Adult | -2.41 | 1.20 | -2.01 | 0.167 |
| 10-11 pd | - | Adult | -1.96 | 0.78 | -2.51 | **0.047** |
| 17-18 pd | - | Adult | -2.99 | 0.87 | -3.44 | **0.002** |
| 26-27 pd | - | Adult | -1.95 | 1.19 | -1.64 | 0.346 |
| END SYLLABLE | | | | | | |
| Cluster 1 (χ² = 10.22; df = 4; p = 0.037) | | | | | | |
| 0-1 pd | - | Adult | 25.00 | 309.00 | 0.08 | 1.000 |
| 10-11 pd | - | Adult | 22.40 | 309.00 | 0.07 | 1.000 |
| 17-18 pd | - | Adult | 22.00 | 309.00 | 0.07 | 1.000 |
| 26-27 pd | - | Adult | 19.90 | 309.00 | 0.06 | 1.000 |
| Cluster 5 (χ² = 14.76; df = 4; p = 0.005) | | | | | | |
| 0-1 pd | - | Adult | -8.72 | 2.47 | -3.54 | **0.002** |
| 10-11 pd | - | Adult | -8.01 | 2.40 | -3.34 | **0.003** |
| 17-18 pd | - | Adult | -7.86 | 2.30 | -3.41 | **0.003** |
| 26-27 pd | - | Adult | -6.10 | 2.75 | -2.21 | 0.103 |

Tab.S5.

Temporal description of the acoustic structure of the calling bouts. SD = standard deviation; n = number of samples; pd = postnatal day.

| Age class | Mean | SD | n |
| --- | --- | --- | --- |
| **Number of syllables** | | | |
| 0-1 pd | 3.79 | 2.89 | 48 |
| 10-11 pd | 3.46 | 2.90 | 112 |
| 17-18 pd | 9.01 | 6.60 | 89 |
| 26-27 pd | 12.41 | 8.56 | 17 |
| Adult | 14.44 | 6.07 | 50 |
| **Intersyllable-interval [ms]** | | | |
| 0-1 pd | 123.22 | 33.49 | 85 |
| 10-11 pd | 72.08 | 35.93 | 165 |
| 17-18 pd | 51.43 | 29.84 | 507 |
| 26-27 pd | 20.93 | 14.00 | 156 |
| Adult | 14.82 | 9.53 | 530 |
| **Duration of first syllable [ms]** | | | |
| 0-1 pd | 64.14 | 29.37 | 21 |
| 10-11 pd | 58.14 | 55.02 | 35 |
| 17-18 pd | 70.75 | 40.70 | 56 |
| 26-27 pd | 87.82 | 43.56 | 11 |
| Adult | 179.80 | 137.99 | 46 |
| **Duration of middle syllable [ms]** | | | |
| 0-1 pd | 70.35 | 22.46 | 20 |
| 10-11 pd | 46.90 | 29.22 | 41 |
| 17-18 pd | 46.05 | 27.57 | 62 |
| 26-27 pd | 26.00 | 22.23 | 12 |
| Adult | 21.38 | 29.72 | 42 |
| **Duration of end syllable [ms]** | | | |
| 0-1 pd | 63.57 | 27.47 | 21 |
| 10-11 pd | 43.74 | 24.16 | 38 |
| 17-18 pd | 40.28 | 23.09 | 58 |
| 26-27 pd | 30.60 | 34.09 | 5 |
| Adult | 15.84 | 3.50 | 38 |

Tab.S6.

Pairwise comparisons between the adult and the infants age classes for the clusters which showed a global significant effect of age class on the temporal characteristics using LMMs. Bold: p<0.05; pd = postnatal day.

| Contrast | | | Estimate | SE | t - ratio | P - value |
| --- | --- | --- | --- | --- | --- | --- |
| Number of syllables (χ² = 79.96; df = 4; p < 0.001) | | | | | | |
| 0-1 pd | - | Adult | -9.73 | 1.47 | -6.63 | **<0.001** |
| 10-11 pd | - | Adult | -10.07 | 1.31 | -7.68 | **<0.001** |
| 17-18 pd | - | Adult | -6.57 | 1.30 | -5.06 | **<0.001** |
| 26-27 pd | - | Adult | -4.07 | 1.67 | -2.44 | 0.061 |
| Intersyllable-interval (χ² = 1159.56; df = 4; p < 0.001) | | | | | | |
| 0-1 pd | - | Adult | 105.00 | 3.50 | 29.98 | **<0.001** |
| 10-11 pd | - | Adult | 61.60 | 2.67 | 23.10 | **<0.001** |
| 17-18 pd | - | Adult | 33.40 | 2.47 | 13.52 | **<0.001** |
| 26-27 pd | - | Adult | 20.10 | 3.18 | 6.31 | **<0.001** |
| Duration of the first syllable (χ² = 22.07; df = 4; p < 0.001) | | | | | | |
| 0-1 pd | - | Adult | -117.70 | 28.50 | -4.13 | **<0.001** |
| 10-11 pd | - | Adult | -110.60 | 25.20 | -4.39 | **<0.001** |
| 17-18 pd | - | Adult | -94.20 | 23.60 | -3.98 | **0.001** |
| 26-27 pd | - | Adult | -81.10 | 32.40 | -2.50 | 0.053 |
| Duration of the middle syllable (χ² = 47.26; df = 4; p < 0.001) | | | | | | |
| 0-1 pd | - | Adult | 54.50 | 8.43 | 6.46 | **<0.001** |
| 10-11 pd | - | Adult | 30.10 | 6.75 | 4.47 | **<0.001** |
| 17-18 pd | - | Adult | 23.40 | 6.25 | 3.75 | **0.001** |
| 26-27 pd | - | Adult | 10.70 | 9.05 | 1.18 | 0.667 |
| Duration of the end syllable (χ² = 78.69; df = 4; p < 0.001) | | | | | | |
| 0-1 pd | - | Adult | 56.70 | 6.70 | 8.47 | **<0.001** |
| 10-11 pd | - | Adult | 31.50 | 5.36 | 5.88 | **<0.001** |
| 17-18 pd | - | Adult | 21.70 | 5.08 | 4.27 | **<0.001** |
| 26-27 pd | - | Adult | 19.10 | 9.29 | 2.06 | 0.155 |

Tab.S7.

Acoustic description (mean ± standard deviation) of the acoustic parameters of Cluster 1 across the age classes. pd = postnatal day.

| Age class | 0-1 pd | 10-11 pd | 17-18 pd | 26-27 pd | Adult |
| --- | --- | --- | --- | --- | --- |
| Temporal parameters | | | | | |
| Syllable duration [ms] | 75.36  ± 19.48 | 62.76  ± 29.09 | 48.80  ± 25.46 | 58.10  ± 45.61 | 33.44  ± 23.50 |
| Time of minimum F0 [ms] | 37.12  ± 26.95 | 23.38  ± 29.23 | 11.54  ± 16.91 | 17.75  ± 33.40 | 6.68  ± 15.08 |
| Time of maximum F0 [ms] | 42.52  ± 35.26 | 49.84  ± 27.44 | 39.27  ± 23.42 | 40.50  ± 32.09 | 25.12  ± 18.49 |
| Spectral parameters | | | | | |
| Minimum F0 [kHz] | 19.43  ± 3.06 | 20.32  ± 2.04 | 20.24  ± 1.53 | 23.25  ± 4.33 | 24.56  ± 3.39 |
| Maximum F0 [kHz] | 21.89  ± 2.78 | 22.03  ± 2.05 | 22.41  ± 2.19 | 28.34  ± 4.24 | 28.25  ± 3.31 |
| Mean F0 [kHz] | 20.32  ± 2.69 | 21.04  ± 2.02 | 21.27  ± 1.80 | 25.71  ± 4.21 | 26.36  ± 3.52 |
| Standard deviation of F0 [kHz] | 0.65  ± 0.34 | 0.43  ± 0.39 | 0.60  ± 0.40 | 1.45  ± 0.79 | 1.22  ± 0.53 |
| Bandwidth [kHz] | 2.46  ± 1.26 | 1.71  ± 1.34 | 2.17  ± 1.31 | 5.09  ± 2.87 | 3.69  ± 1.65 |
| Mean slope [kHz/s] | 54.16  ± 23.01 | 94.98  ± 114.42 | 136.14  ± 96.88 | 339.60  ± 176.69 | 294.70  ± 277.21 |

Tab.S8.

Acoustic description (mean ± standard deviation) of the acoustic parameters of Cluster 3 across the age classes. pd = postnatal day.

| Age class | 0-1 pd | 10-11 pd | 17-18 pd | 26-27 pd | Adult |
| --- | --- | --- | --- | --- | --- |
| Temporal parameters | | | | | |
| Syllable duration [ms] | 16.33  ± 2.08 | 21.59  ± 14.34 | 24.21  ± 20.13 | 28.96  ± 23.86 | 21.78  ± 17.06 |
| Time of minimum F0 [ms] | 13.67  ± 2.31 | 8.38  ± 10.69 | 5.63  ± 12.86 | 12.07  ± 22.51 | 4.55  ± 12.19 |
| Time of maximum F0 [ms] | 2.00  ± 0.00 | 11.25  ± 9.78 | 16.56  ± 11.58 | 17.43  ± 10.88 | 15.34  ± 10.66 |
| Spectral parameters | | | | | |
| Minimum F0 [kHz] | 22.22  ± 2.69 | 17.67  ± 3.92 | 17.23  ± 3.63 | 16.82  ± 3.81 | 16.09  ± 2.57 |
| Maximum F0 [kHz] | 38.89  ± 2.62 | 29.14  ± 5.26 | 26.88  ± 3.68 | 27.33  ± 2.79 | 25.78  ± 2.94 |
| Mean F0 [kHz] | 29.83  ± 3.71 | 23.73  ± 3.64 | 23.01  ± 3.37 | 23.04  ± 3.07 | 21.46  ± 2.61 |
| Standard deviation of F0 [kHz] | 5.62  ± 0.64 | 3.85  ± 1.54 | 3.25  ± 0.74 | 3.59  ± 1.01 | 3.49  ± 0.79 |
| Bandwidth [kHz] | 16.68  ± 1.50 | 11.47  ± 5.08 | 9.65  ± 2.53 | 10.51  ± 3.22 | 9.69  ± 2.27 |
| Mean slope [kHz/s] | 1453.34  ± 182.20 | 858.36  ± 315.28 | 746.28  ± 192.65 | 730.55  ± 154.35 | 808.95  ± 173.27 |

Tab.S9.

Acoustic description (mean ± standard deviation) of the acoustic parameters of Cluster 5 across the age classes. pd = postnatal day.

| Age class | 0-1 pd | 10-11 pd | 17-18 pd | 26-27 pd | Adult |
| --- | --- | --- | --- | --- | --- |
| Temporal parameters | | | | | |
| Syllable duration [ms] | 43.29  ± 19.14 | 26.26  ± 13.58 | 16.65  ± 8.02 | 15.11  ± 4.33 | 15.50  ± 5.08 |
| Time of minimum F0 [ms] | 8.06  ± 9.91 | 9.17  ± 9.54 | 2.43  ± 1.28 | 2.92  ± 3.74 | 2.52  ± 2.83 |
| Time of maximum F0 [ms] | 33.00  ± 23.82 | 17.05  ± 16.31 | 13.12  ± 8.09 | 10.00  ± 3.23 | 11.27  ± 3.98 |
| Spectral parameters | | | | | |
| Minimum F0 [kHz] | 11.30  ± 2.80 | 11.46  ± 4.69 | 15.64  ± 2.49 | 13.91  ± 2.61 | 14.03  ± 1.86 |
| Maximum F0 [kHz] | 14.50  ± 4.32 | 14.79  ± 4.93 | 19.77  ± 2.98 | 18.22  ± 3.30 | 18.08  ± 2.86 |
| Mean F0 [kHz] | 12.97  ± 3.40 | 13.18  ± 4.79 | 17.95  ± 2.80 | 16.32  ± 2.94 | 16.24  ± 2.27 |
| Standard deviation of F0 [kHz] | 0.93  ± 0.73 | 1.09  ± 0.48 | 1.45  ± 0.74 | 1.56  ± 0.73 | 1.47  ± 0.76 |
| Bandwidth [kHz] | 3.21  ± 2.33 | 3.33  ± 1.37 | 4.13  ± 1.93 | 4.32  ± 2.01 | 4.05  ± 2.07 |
| Mean slope [kHz/s] | 123.84  ± 70.30 | 248.87  ± 193.95 | 530.13  ± 304.49 | 493.03  ± 242.41 | 442.81  ±225.69 |

Tab.S10.

Results of the LMMs testing effects of age class on the acoustic parameters of Cluster 1, 3 and 5. Bold: p <0.05; **↗ = increasing with age;** ↘ = decreasing with age.

| Parameters | Cluster 1 (df = 4) | | | Cluster 3 (df = 4) | | | Cluster 5 (df = 4) | | |
| --- | --- | --- | --- | --- | --- | --- | --- | --- | --- |
|  | χ² | P - value | Dir. | χ² | P - value | Dir. | χ² | P - value | Dir. |
| Temporal parameters | | | | | | | | | |
| Syllable duration  [ms] | 31.11 | **<0.001** | ↘ | 3.90 | 0.419 |  | 173.04 | **<0.001** | ↘ |
| Time of minimum F0  [ms] | 74.90 | **<0.001** | ↘ | 6.95 | 0.139 |  | 54.32 | **<0.001** | ↘ |
| Time of maximum F0  [ms] | 12.05 | **0.020** | ↘ | 4.29 | 0.368 |  | 135.69 | **<0.001** | ↘ |
| Spectral parameters | | | | | | | | | |
| Minimum F0  [kHz] | 42.27 | **<0.001** | **↗** | 23.84 | **<0.001** | ↘ | 59.22 | **<0.001** | **↗** |
| Maximum F0  [kHz] | 72.74 | **<0.001** | **↗** | 88.39 | **<0.001** | ↘ | 29.08 | **<0.001** | **↗** |
| Mean F0  [kHz] | 57.61 | **<0.001** | **↗** | 43.37 | **<0.001** | ↘ | 39.96 | **<0.001** | **↗** |
| Standard deviation of F0  [kHz] | 60.20 | **<0.001** | **↗** | 33.75 | **<0.001** | ↘ | 11.36 | **0.023** | **↗** |
| Bandwidth [kHz] | 56.62 | **<0.001** | **↗** | 31.94 | **<0.001** | ↘ | 5.41 | 0.247 |  |
| Mean slope [kHz/s] | 69.03 | **<0.001** | **↗** | 41.60 | **<0.001** | ↘ | 39.50 | **<0.001** | **↗** |

Tab.S11.

Recapitulation of the features of human babbling defined in this study referring to the features introduced by Elowson et al.*^10^* and Fernandez et al.*^11^*, as well as the features of animal babbling described for marmosets, bats and gray mouse lemurs. In the latter case this is based on the findings of this study, with the exception of the feature marked in gray, which was based on findings by Scheumann et al.*^30^*.

| **Human babbling features** | **Elowson** | **Fernandez** | **Marmosets** | **Bats** | **Gray mouse lemurs** |
| --- | --- | --- | --- | --- | --- |
| **Universality**  i.e., protophones produced by infants irrespective of their cultural background | **Universality** | **Universality** | **Yes,**  all infants babbled | **Yes,**  all pups of two populations babbled | **Yes,**  clusters were produced by several different infants |
| **Syllable subset acquisition**  i.e., includes phonetic units found in adult speech | **Subset of phonetic units** | **Syllable subset acquisition** | **Yes,**  not all adult call types were produced by infants | **Yes**,  pups acquired 12-20 of the 25 adult syllable types | **Yes,**  Infants produce Cluster 1, 2, 3 and 5 of the adult trill; Trills as a call type not present at birth. |
| **Independence of social context**  i.e., occurs also in the absence of a vocal referent and lacks apparent meaning | **No meaning** | **Nonmandatory social context** **and function** | **Yes**,  babbling irrespective of context, also observed as a monologue | **Yes**,  babbling self-initiative, does not always occur before nursing | **Yes,**  produced irrespective of context and social referent |
| **Rhythmic and Repetitive**  i.e., rhythmic phonation and repetition of canonical syllables | **Rhythmic and Repetitive** | **Rhythmicity** and **Reduplication** | **Yes**,  call types were repeated within sequences | **Yes,**  syllables self-repeating and produced in bouts with a regular beat | **Yes,**  syllables produced in bouts, self-loops and repetition of different syllable types |
| **Babbling bout composition**  i.e., combination of consonant and vowel units into sequences | **Consonant-vowel structure** | **Babbling bout composition** | **Yes,**  call types combined thus resembling adult sequences | **Yes,**  proto-syllables combined with adult-like syllables | **Yes,**  increased combination of syllable types, gradually resembling adult trill call |
| **Peak of high vocal plasticity during infancy**  i.e., early onset of babbling with a later peak of emergence of syllable types | **Peak of high plasticity** | **Syllable type emergence** and **Early babbling onset** | **Partly,**  starts during early infancy but prolonged vocal plasticity | **Yes,**  syllable acquisition nonlinear, sudden expansion of syllable types | **Weak evidence**,  starts during early infancy, expansion of two additional syllable types |
| **Facilitating caregiver interactions**  i.e., used during oscillating parent-infant interactions | **Caregiver-child bond** | – | **Yes**,  uttered during infant-caregiver interactions | **Yes**,  uttered during mother-pup interactions | **Yes,**  used in mother-infant greeting context |

Tab.S12.

Overview of the number of calling bouts and syllables in the analysis for the infants in each age class for the different isolation contexts. In natural isolation the infants were recorded in the nest box while the mother was outside foraging. During experimental isolation context, the infants were separated from their mother and siblings and placed for 5 minutes in a sound-damped chamber or isolated infants responded to playbacks of adult trill calls. pd = postnatal day.

| Infant age class | Experimental paradigm | Number of calling bouts | Number of syllables |
| --- | --- | --- | --- |
| 0-1 pd | Natural isolation context | 48 | 141 |
|  | Experimental isolation context | 0 | 0 |
| 10-11 pd | Natural isolation context | 76 | 171 |
|  | Experimental isolation context | 36 | 123 |
| 17-18 pd | Natural isolation context | 28 | 236 |
|  | Experimental isolation context | 61 | 387 |
| 26-27 pd | Natural isolation context | 15 | 176 |
|  | Experimental isolation context | 2 | 4 |

Tab.S13.

Overview of the audio recordings used in this study, the experimental conditions and the recording equipment. Natural Isolation: Infants were recorded in the nest box while the mother was outside foraging. Experimental isolation context: Infants were separated from their mother and siblings and placed for 5 minutes in a sound-damped chamber or isolated infants responded to playbacks of adult trill calls. Social encounter paradigm: Two subjects (same sex or opposite sex) were positioned in two test cages, which were connected by a door. Animals were observed for 30 minutes with the door closed and 30 minutes with an open door. Mother-infant reunion: An infant was placed in a test cage and the mother was released. The experiment lasted until the mother carried the baby back to the nest or for a maximum of 10 minutes. Playback: A subject was placed in a test cage and calls from conspecifics, heterospecifics, predators or infants were played back from a loudspeaker. MFR = microphone frequency response.

| Age group | Experimental paradigm | Recording equipment |
| --- | --- | --- |
| Infants | Natural isolation context | Song Meter SM2 Bat + Wildlife Acoustic recorder (16-bit. 192 kHz sampling rate); MFR (SMX-II): 15 to 40 kHz ± 5 dB |
|  | Experimental isolation context^1.2^ | ^1^Pettersson D1000X ultrasound detector (16-bit. 250 kHz sampling rate); MFR: 5-235 kHz |
|  |  | ^2^U30 Batdetector via control-filter unit (Pettersonbox F2000) linkted to an analog/digital converter (PCM-DAS 16/330) in a laptop (12-bit. 200 kHz); MFR: 8–100 kHz |
| Adults | Social encounters^1.2.3^  Mother-infant reunions^1.2.3^  Playback of infant calls^1.2.3^  Playback of conspecific and heterospecific calls^3^ | ^1^Pettersson D1000X ultrasound detector (16-bit. 250 kHz sampling rate); MFR: 5-235 kHz |
|  |  | ^2^U30 Batdetector via analog/digital filter (DAQCard-6062E) linked to a computer (12-bit. 200-500 kHz sampling rate); MFR: 8–100 kHz |
|  |  | ^3^U30 Batdetector via control-filter unit (Pettersonbox F2000) linkted to an analog/digital converter (PCM-DAS 16/330) in a laptop (12-bit. 200 kHz); MFR: 8–100 kHz |
